# Relationship between Parenting and Cognitive Schemas in a Group of Male Adult Offenders

**DOI:** 10.3389/fpsyg.2016.00302

**Published:** 2016-03-07

**Authors:** Monica Pellerone, Giuseppe Craparo, Ylenia Tornabuoni

**Affiliations:** Faculty of Human and Social Sciences, “Kore” University of EnnaEnna, Italy

**Keywords:** parenting, attachment style, cognitive schemas, behavioral responses, offenders

## Abstract

This work analyzes the correlation of retrospective ratings on parental binding with cognitive patterns in the inmates for property crimes. The participant group comprehended 248 adults men, including 130 marked out as offenders (the target group), aged between 19 and 70, currently serving sentences in the Cavadonna prison in Siracusa, and 118 marked out as non-offenders (the control group), aged between 20 and 70, living in Siracusa (Sicily). The instruments used were the Parental Bonding Instrument (PBI), and the Young Schema Questionnaire-3 (YSQ). The preliminary analysis showed a high percentage of offenders who experienced an affectionate constraint parenting. Offenders scored significantly higher than the non-offenders on the level of paternal control and the YSQ subscales. The study underlines the influence of maternal care on most of the cognitive schemas, and the role of father's control on the tendency to social isolation and defectiveness in the offenders.

## Introduction

The cognitive model investigates the beliefs and cognitive schemas that determine the affective responses and behavioral strategies of the individual (Beck et al., 1990); beliefs or schemas are the cognitive representations of life experiences; in some cases, these experiences can lead to identity crisis (Pellerone et al., 2015), maladaptive defense mechanism, dissociated emotions (Craparo et al., 2014a,b; Gori et al., 2014), maladaptive beliefs and behaviors (McMurrana and Christopherb, 2008).

This model is based on the assumption that environmental stimuli associated with a personal elaboration of their meaning, arouse a subsequent physiological and emotional response. These emotions, in turn, have a powerful reciprocal effect on cognitive content, stimulating dysfunctional thoughts. Thus, behavioral responses to stimuli could be the result but also the cause of maladaptive cognitions (Wright et al., 2003; Friedman and Thase, 2006; Friedman et al., 2008; Craparo et al., 2014c).

Jeffrey Young's model underlines that individuals with complex problems have one or more early maladaptive schemas, which make them vulnerable to emotional disorders. An early maladaptive schema has been defined as a broad pervasive theme or pattern regarding oneself and one's relationship with others, developed during childhood and elaborated throughout one's lifetime. Schemas are extremely stable and enduring patterns, comprised of memories, corporeal sensations, emotions, cognitions, and once they are activated intense emotions are felt.

The author identified 18 early maladaptive schemas that have been organized into five themes known as domains:

- *disconnection and rejection domain:* people with these schemas expect that their needs for stability, nurturance, security, and empathy in family relationships will not be met in a consistent or predictable way;
- *impaired autonomy and performance*: this domain has to do with expectations about oneself and the environment that interfere with one's ability to separate and function independently and one's perceived ability to survive alone; typically the family of origin is enmeshed or overprotective;
- *impaired limits domain*: characterized by deficiencies in internal limits, respect, and responsibility to others; typically the family of origin is characterized by permissiveness and indulgence;
- *other-directedness domain:* relates to an excessive focus on meeting the needs of others, at the expense of one's own needs; Typically the family of origin gave only conditional acceptance, resulting in the children suppressing normal needs and emotions in order to gain attention and approval;
- *overvigilance and inhibition domain*: characterized by an excessive focus on controlling, suppressing, or ignoring of one's emotions; typical of the family of origin are domination and suppression of feelings, or a bleak environment where performance standards and self-control take priority over pleasure and playfulness.

The literature underlines the presence of a connection between maladaptive cognitive schemas and attitudes in favor of crime; for example criminological thought leads to the reiteration of dysfunctional schemas and antisocial behaviors in both criminals and non-criminal subjects. In the non-offenders these thought patterns may not lead to illegal behavior, but to irresponsible or maladaptive consequences; in contrast, in convicted criminals these mental processes may determine further antisocial and illegal behavior (Baker and Beech, 2004; Chakhssi et al., 2013; Gonzalez et al., 2013).

The literature also shows that among the predictors of criminological thought there are: the criminological needs, the criminal history, or the history of antisocial behavior (Anderson and Bushman, 2002), individual factors such as age and sex, but especially family characteristics, such as parenting education and parenting styles; less robust predictors are the intellectual functioning (Gendreau et al., 1996), factors of personal danger and the socio-economic status of the family of origin (Mandracchia and Morgan, 2012).

In fact, in early childhood parent-child interactions may be partly responsible for the development of criminological thought and the parenting style of both parents may be linked to delinquent behavior in children: the recent literature shows, that negligent parenting is correlated to high levels of delinquency in males and the presence of permissive parents is related to delinquent behavior in females. Moreover, it would seem that the levels of crime are lower in families with at least one authoritative parent and are highest in families where both parents are negligent (Hoeve et al., 2009), because social and parental support are related to a greater psychological adjustment (Pellerone and Miccichè, 2015).

Violence is the product of a chain of events over the course of a child's development, where risks accumulate and reinforce each other (Maughan and Rutter, 2001).

Literature exploring the background of detainees identifies their motivation as not only economic but also the result of frustration, disregard for human life, and lack of family cohesion. More destructive young people, often, come from numerous families with absent parents, who live in conditions of emotional-affective deprivation and poor supervision (Farrington, 1995, 2003). Disruptive family backgrounds including childhood separation and trauma are common in the history of offenders and delinquents (Bowlby, 1944, 1988; Lewis, 1989).

On the other hand, sufficient parental monitoring and a good level of parental support for children are related to low levels of delinquent behavior in adolescence (Caldwell et al., 2006; Noe Sean, 2008; Pellerone, 2013).

Bowlby (1944) suggests that deprivation disrupts early attachment relationships and causes children to seek self-protection by avoiding or dismissing attachment relationships, and as a result they do not have a way of forestalling the emergence of delinquency in the context of other personal and environmental risk factors (Rutter et al., 1998; Rutter, 2000). The avoidance of attachment relationships may also be directly involved in the etiology of conduct disorder (Greenberg et al., 1993; Fonagy and Levinson, 2004), which is a risk factor for offending.

So, the emotional relationship between parents and children could influence all dimensions of habitual delinquency, both at the cognitive and the behavioral levels (Barnes and Farrell, 1992; Biggam and Power, 1998; Juang and Silbereisen, 1999). In this regard, Aime (2008) investigating the role of attachment (in terms of care and protection) on dysfunctional patterns in a group of incarcerated women (some of them victims of childhood sexual abuse) shows that: women who have experienced an attachment characterized by low nurturance have more severe dysfunctional schemas; and women with an attachment characterized by a high level of overprotection have higher dysfunctional schemas, than women with low levels of overprotection. The author concluded that although childhood sexual abuse is related to the development of cognitive schemas, attachment appears to be the most important predictor of cognitive dysfunctional patterns.

Similarly Smallbone and Dadds (1998), investigating the role of attachment in non-offenders, sex-offenders, and detainees imprisoned for property crimes, have shown how: the group of sex-offenders reports an infant attachment and an adult parenting significantly less safe than the non-offenders; moreover, the group of sex-offenders has a maternal attachment that is less secure than those imprisoned for property crimes.

Based on these results, it seems essential to further explore the correlation of retrospective ratings on parental binding with cognitive patterns in the inmates (Chambers et al., 2001; Thimm, 2013); toward this end, the study compares scores between offenders grown-ups and grown-ups with similar socio-demographic characteristics who have not committed offenses throughout their lives. The objectives of this work are:

- to investigate the presence of cognitive schemas, classified according to Young's model (1990), in a group of male adult prisoners; in particular, it is assumed the presence of maladaptive schemas such as social hostility and rejection, tendency to over-vigilance, and self-injurious behaviors, an hypothesis which accords with the international literature (Baker and Beech, 2004; Chakhssi et al., 2013; Gonzalez et al., 2013).
- to explore whether parental style influences the cognitive patterns in a group of offenders; hypothesizing that prisoners who experienced a dysfunctional attachment with their mother, characterized by low care, show more severe cognitive schemas, such as the tendency to social isolation, the perception of emotional deprivation, abandonment, and vulnerability (Smallbone and Dadds, 1998; Aime, 2008).
- to investigate the predictive variables of these cognitive schemas in adult offenders, assuming that the condition of detainee, together with a low level of parental care and high level of parental control (Parker et al., 1979; Burton et al., 1995), can be predictive variables on the development of severe cognitive schemas (Chambers et al., 2001).

## Materials and methods

### Subjects

The research project involved 248 male adult: the target group was formed of 130 male prisoners, aged between 19 and 70 (*M* = 35.42; *SD* = 11.68), with antisocial personality disorder (pure ASPD); this diagnosis had been made during the prisoners' induction phase by the prison psychiatrist who administered the SCID II (First et al., 1997). The control group was composed of 118 healthy adult man, who had not committed offenses, aged between 20 and 70 (*M* = 36.59, *SD* = 9.57).

The target group' educational levels were as follows: 73.6% had a middle school diploma; 13.8% had a high school diploma, only 1.5% had a bachelor's degree, and 1.5% did not report their education. The control group' educational levels were as follows: 73.7% had a middle school diploma; 14.4% had a high school diploma, 1.7% had a bachelor's degree.

No statistical differences were detected between the target and control groups based on age (χ^2^ = 938.14; *p* = 0.27) and education levels (χ^2^ = 6.49; *p* = 0.89).

Eligible prisoners were identified by a member of prison staff and invited to volunteer. This group was recruited from a prison in Sicily. Adult male prisoners convicted for only minor crimes, of a non-sexual nature, were eligible for the study, unless excluded by the following criteria. First, those with < 4 months to discharge were excluded, so that all recruits would have enough time to complete the study. Second, those who had taken part in intervention programs within the 6 months prior to recruitment were excluded. A prison liaison person recruited potential participants by informing prisoners; volunteers were then seen by the researcher, who gave further information about the study.

Ethical approval for the study was obtained from the relevant ethics committee, and all participants signed statements of their informed consent which were then approved by the ethics committee. All subjects were informed that participation was not paid, and they were made aware that they could withdraw from the study at any time.

### Measures

The participants completed the Structured Clinical Interview for DSM-IV Axis II Personality Disorders, the Parental Bonding Instrument and the Young Schema Questionnaire.

The Structured Clinical Interview for DSM-IV Axis II Personality Disorders (SCID-II; First et al., 1997) is an interview used to diagnose a number of personality disorders, such as: avoidant, dependent, obsessive-compulsive, passive-aggressive, depressive, paranoid, schizotypal, schizoid, histrionic, narcissistic, borderline, and antisocial personality disorder. In this Interview a score of 1 is assigned if the trait is not present, 2 is assigned if the trait is present, but not clinically significant, and 3 is assigned when the trait is present and clinically relevant (Mazzi et al., 2003). Diagnosis is based on the specific cut-off scores (see Table 1).

**Table 1:** **Means and standard deviation of SCID II and cut off scores**.

**Scales**	**Participants**		**Cut off scores**
	***M***	***SD***	
Avoidant Personality	1.610	0.907	4
Dependent Personality	2.015	1.057	5
Obsessive-Compulsive Personality	1.460	0.497	4
Passive-Aggressive Personality	2.309	1.120	4
Depressive Personality	1.859	0.867	5
Paranoid Personality	1.920	0.603	4
Schizotypal Personality	1.345	0.963	5
Schizoid Personality	1.210	0.633	4
Histrionic Personality	2.313	1.060	5
Narcissistic Personality	2.384	0.240	5
Borderline Personality	2.435	0.850	5
Antisocial Personality	3.384	1.130	3

The Parental Bonding Instrument (PBI; Parker et al., 1979) is a questionnaire consisting of 25 items, divided into two parts (one for the mother and one for the father), which measures the perception of behavior of the parents during childhood. The measure is retrospective, meaning that adults (over 16 years) complete the measure for how they remember their parents during their first 16 years. The instrument investigates the processes of parenting across two domains, parental care and control or overprotection, from the combination of which the authors classify four types of attachment: (a) *affectionate constraint*: high scores both in the scale care and overprotection; (b) *optimal parenting*: high care and low protection; (c) *affectionless control*: high protection and low care; (d) *neglectful parenting*: low care and low protection (Parker, 1989).

Assignment to “high” or “low” categories of domains is based on the following cut-off scores: for mothers, a care score of 27.0 and a protection score of 13.5 are the minimum scores for inclusion in the “high” category; for fathers, a care score of 24.0 and a protection score of 12.5 are the cut off points. In the Italian adaptation, Picardi and collaborators report the estimates of internal consistency of the tool: 0.75 for the results that indicate mother's care, 0.84 for the scores that indicate mother's overprotection; 0.83 for father's care, and 0.88 for father's overprotection (Picardi et al., 2005).

The Young Schema Questionnaire-3 (YSQ-3; Young, 2005) is a questionnaire consisting of 232 items, which investigate 18 cognitive patterns, grouped into five domains, namely:

- *disconnection and rejection domain:* emotional deprivation, abandonment, mistrust or abuse, social isolation, and defectiveness or shame;
- *impaired autonomy and performance domain:* failure to achieve, dependence on others, vulnerability to harm, and relational enmeshment;
- *other-directedness domain:* subjugation, self-sacrifice; recognition seeking;
- *overvigilance and inhibition domain:* emotional inhibition, unrelenting standards, pessimism, and punitiveness;
- *impaired limits domain:* entitlement or superiority and insufficient self-control.

The long form of the Italian version was used, and all 18 schemas were examined (Saggino et al., 2014). Assignment to “high” or “low” categories of domains is based on the following cut-off scores: on the long form, any score of 3 or more is considered meaningful (both single score of each schema, that composite score of each domain); on the short form, any score of 2 or more is usually meaningful (Young et al., 2007).

### Analysis methods

All analyses were conducted with SPSS 19.0.

In the target group, the descriptive statistic was used to assess the type of parental style; the Rho Pearson correlation was conducted in order to investigate the relation between cognitive schemas; two univariate analyses of variance (ANOVA one-way) were used to measure the influence of independent variables (age and education level) on schemas.

The descriptive statistic was used to assess the first research objective, that is the presence of dysfunctional cognitive patterns, comparing mean scores of people detained, of the control group and cut-off scores. Student's *t*-test for independent samples was used to compare the mean between groups (offenders vs. control).

In both groups, two multivariate analyses of variance (MANOVA) were conducted to investigate the second research objective, that is the influence of parenting on cognitive schemas in adulthood, respectively, one for maternal parenting, and one for paternal parenting.

An analysis of hierarchical regression for separate blocks was used to detect the last research objective, that is the presence of predictor variables of dysfunctional cognitive schemas: (a) condition of detention and non-detention in the 1st block; (b) age and education level in the 2nd block; (c) mother's parenting (care and over-control) in the 3rd block; (c) father's parenting (care and overprotection) in the 4th block. This multiple regression analysis was used because of its great suitability to testing the direct effect of parenting style (the predictor variable) on cognitive schema (the dependent variable). The use of this model has been confirmed primarily by Young et al. (2003), who emphasized the role of parents in the development of the Early Maladaptive schemas (direct effect of a predictor variable on a dependent variable) and not *vice-versa*; also, recent literature emphasizes the use of such a model to assess the direct effect of parenting style on dysfunctional cognitive schemas of prisoners (Biggam and Power, 1998; Aime, 2008; Alfasfos, 2009).

## Results

### Descriptive statistics

A descriptive statistical analysis was conducted in order to investigate the mother's parenting. The results show that 29% of the offenders had an affectionate constraint attachment, characterized by high level of care and control; followed by 25% with optimal parenting (high nurturance and low overprotection); 23% with affectionless control (low nurturance and high control), and 22% with neglectful parenting (low nurturance and low control). The same analysis of the data was conducted on the father's parenting: results show that 28% of the offenders had an affectionate constraint, followed by 25% with negligent attachment, another 24% had an optimal parenting.

*t*-Test shows, that the offenders reported average scores significantly higher than the non-offenders in the level of paternal control [*t*_(242, 51)_ = 22.64, *p* < 0.001].

The Rho Pearson correlation analysis was conducted in order to investigate the relation between cognitive schemas in the offenders; it showed how all the domains are related to each other (see Table 2).

**Table 2:** **Pearson's correlation for scores of cognitive schemas in the target group**.

**Measures**	**a**.	**b**.	**c**.	**d**.	**e**.
a. Rejection	–				
b. Impaired autonomy	0.782^**^	–			
c. Other-directedness	0.677^**^	0.821^**^	–		
d. Over-vigilance	0.598^**^	0.750^**^	0.792^**^	–	
e. Impaired limit	0.487^**^	0.619^**^	0.690^**^	0.828^**^	–

** *Correlation is significant at the 0.01 level (two-tailed)*.

The univariate analysis of variance (ANOVA one-way) was also performed to test the influence of age (independent variables) on cognitive schemas in the target group, showing how age affects only the sense of entitlement [*F*_(1, 129)_ = 3.97, *p* < 0.05]: the analysis of the average scores demonstrates that the group of younger inmates presents higher scores (*M* = 2.88; *SD* = 1.24) than the mature adults (*M* = 2.48; *SD* = 0.99). The same analysis of the data shows that the level of education does not affect cognitive schemes (*p* > 0.05).

A descriptive analysis was conducted in order to investigate the cognitive patterns, comparing mean scores of people detained, the control group, and the cut-off scores (for the Italian population). It shows that (see Table 3): in reference to the target group, in the *disconnection and rejection domain* high scores emerge in the schema of mistrust and the fear of being abandoned; in the *over-vigilance domain*, the participants get higher scores in unrelenting standards; in the *other-directedness domain*, the highest value is that of the tendency to sacrifice for others. The research hypothesis seems therefore to be partially confirmed. In the control group, all schemes have lower scores than cut off scores.

**Table 3:** **Means and standard deviations of YSQ-3 subscales in all participants**.

**Subscales**	**Target Group**		**Control Group**		**Levene's Test**		**Student's Test**		
	***M***	***SD***	***M***	***SD***	***F***	***P*-value**	***t***	***df***	***p***
**DISCONNECTION AND REJECTION DOMAIN**									
Emotional deprivation	2.610	1.307	2.093	0.864	21.678	<0.001	3.636	246	<0.001
Abandonment	3.015	1,057	2.423	0.850	5.158	0.024	4.830	246	<0.001
Mistrust	6.460	1.949	2.724	1.149	23.477	<0.001	18.154	246	<0.001
Social isolation	2.309	1.110	1.862	0.690	15.346	<0.001	3.768	246	<0.001
Defectiveness	1.859	0.867	1.778	0.683	2.927	0.088	0.809	246	0.419
**IMPAIRED AUTONOMY AND PERFORMANCE DOMAIN**									
Failure	1.920	1.103	1.812	0.850	3.733	0.050	0.855	246	0.393
Dependence	1.945	0.963	1.838	0.833	1.845	1.176	0.931	246	0.353
Vulnerability	2.610	1.233	2.382	1.040	2.570	0.110	1.572	246	0.117
Enmeshment	2.313	1.060	2.044	0.799	9.980	0.002	2.231	246	0.027
**OTHER-DIRECTEDNESS DOMAIN**									
Subjugation	2.384	1.130	2.129	0.742	11.217	0.001	2.078	246	0.039
Self-sacrifice	3.699	1.263	2.998	0.6777	20.394	<0.001	5.359	246	<0.001
Recognition seeking	2.348	1.177	2.167	0.809	11.709	0.001	1.393	246	0.165
**OVERVIGILANCE AND INHIBITION DOMAIN**									
Emotional inhibition	2.583	1.293	2.489	1.110	1.209	0.273	0.614	246	0.540
Unrelenting standards	3.140	1.274	2.950	0.698	23.858	<0.001	1.434	246	0.153
Pessimism	2.710	1.280	2.487	1.040	2.064	0.152	1.502	246	0.134
Punitiveness	2.901	1.160	2.835	0.929	0.862	0.354	0.491	246	0.624
**IMPAIRED LIMITS DOMAIN**									
Entitlement	2.699	1.149	2.873	0.968	1.993	0.159	-1.277	246	0.203
Insufficient self-control	2.388	1.142	2.409	0.847	5.960	0.015	-0.169	246	0.866

*p < 0.001, two-tailed; p < 0.01, two-tailed; p < 0.05, two-tailed; The assignment to “high” or “low” categories of domains is based on the cut-off score of 3 or more (both single score of each schema, that composite score of each domain)*.

*t*-Test shows, that in almost all cognitive patterns the target group achieved average scores significantly higher than the control group; the difference was not significant only for the following patterns: defectiveness, dependence, vulnerability, pessimism, punitiveness, and entitlements (see Table 3).

### Effects of parenting on cognitive schema

The following analysis was done to investigate the influence of types of maternal parenting on cognitive patterns: a factorial design of four types (affectionate constraint, optimal, affectionless control and neglectful parenting) × 18 (cognitive schemas).

In reference to the target group, MANOVA emphasizes a main effect linked to maternal parenting (Wilks's Lambda = 1.44; *p* < 0.03). The breakdown of the univariate effects (see Table 4), means, and standard deviation of dependent variables (see Table 5) show that: the affectionless control maternal parenting (characterized by a high level of protection and a low level of care) influences the tendency to social isolation, defectiveness, failure, dependence, enmeshment, and subjugation, but above all the perception of emotional deprivation, abandonment, mistrust, vulnerability, that reach dysfunctional scores (cut-off).

**Table 4:** **The breakdown of the univariate effects with respect to the type of maternal parenting in the target group**.

**Measures**	***F***	***P-values***
Emotional deprivation	9.568	<0.001
Abandonment	5.008	0.003
Mistrust	5.289	0.002
Social isolation	3.791	0.012
Defectiveness	7.066	<0.001
Failure	3.931	0.010
Dependence	5.119	0.002
Vulnerability	4.295	0.006
Enmeshment	2.688	0.049
Subjugation	4.463	0.005
Self-sacrifice	0.894	0.446
Recognition seeking	2.197	0.092
Emotional inhibition	1.262	0.290
Unrelenting standards	2.012	0.116
Pessimism	1.618	0.189
Punitiveness	2.670	0.050
Entitlement	1.469	0.226
Insufficient self-control	2.035	0.112

*p < 0.001, two-tailed; p < 0.01, two-tailed; p < 0.05, two-tailed*.

**Table 5:** **Means and standard deviation of cognitive schemas with respect to the type of maternal parenting in the target group**.

**Measures**	**Affectionate constraint**		**Optimal parenting**		**Affectionless control**		**Neglectful parenting**	
	***M***	***SD***	***M***	***SD***	***M***	***SD***	***M***	***SD***
Emotional deprivation	2.119	1.152	2.087	0.883	3.338	1.187	3.095	1.520
Abandonment	2.823	1.053	2.704	0.759	3.614	1.017	2.998	1.183
Mistrust	6.025	2.035	5.781	1.385	7.452	1.798	6.775	2.126
Social isolation	2.100	1.124	1.967	0.864	2.783	0.988	2.483	1.292
Defectiveness	1.514	0.777	1.685	0.662	2.378	0.896	1.970	0.913
Failure	1.643	0.973	1.667	0.881	2.437	1.195	2.034	1.223
Dependence	1.677	0.950	1.717	0.730	2.476	1.025	2.007	0.958
Vulnerability	2.283	1.196	2.376	1.075	3.250	1.259	2.647	1.227
Enmeshment	2.215	1.188	2.151	0.891	2.782	0.996	2.138	1.028
Subjugation	2.095	1.161	2.091	0.763	2.940	1.127	2.521	1.248
Self-sacrifice	3.562	1.593	3.531	1.086	3.986	1.162	3.775	1.039
Recognition seeking	2.162	1.205	2.119	0.996	2.852	1.208	2.330	1.201
Emotional inhibition	2.336	1.306	2.353	1.044	3.041	1.335	2.697	1.404
Unrelenting standards	2.743	1.447	3.169	1.004	3.329	1.369	3.433	1.130
Pessimism	2.466	1.409	2.567	1.059	3.067	1.183	2.828	1.390
Punitiveness	2.643	1.298	2.775	0.960	3.302	0.997	2.968	1.265
Entitlement	2.435	1.257	2.691	0.998	2.967	1.105	2.777	1.189
Insufficient self-control	2.084	1.208	2.392	0.914	2.673	1.196	2.487	1.192

Compared to the level of maternal parenting, a subsequent MANOVA analysis shows the influence of the level of care on most of the cognitive schemas. The breakdown of the univariate effects (see Table 6), means, and standard deviation of dependent variables (see Table 7) show that: low maternal care influences the tendency to social isolation, defectiveness, failure, dependence, subjugation, and recognition seeking; but above all the perception of emotional deprivation, abandonment, mistrust, vulnerability, emotional inhibition, and punitiveness, that reach dysfunctional scores.

**Table 6:** **The breakdown of the univariate effects with respect to the level of maternal care in the target group**.

**Measures**	***F***	***P-value***
Emotional deprivation	27.969	<0.000
Abandonment	9.247	0.003
Mistrust	13.634	<0.000
Social isolation	9.998	0.002
Defectiveness	16.117	<0.000
Failure	9.523	0.002
Dependence	11.244	0.001
Vulnerability	8.713	0.004
Enmeshment	2.276	0.134
Subjugation	11.051	0.001
Self-sacrifice	2.244	0.137
Recognition seeking	4.897	0.029
Emotional inhibition	5.430	0.021
Unrelenting standards	3.665	0.058
Pessimism	3.671	0.058
Punitiveness	4.448	0.037
Entitlement	2.333	0.129
Insufficient self-control	2.931	0.089

**Table 7:** **Means and standard deviation of cognitive schemas with respect to the level of maternal care in the target group**.

**Measures**	**Low level of care**		**High level of care**	
	***M***	***SD***	***M***	***SD***
Emotional deprivation	3.219	1.354	2.105	1.028
Abandonment	3.311	1.136	2.768	0.924
Mistrust	7.119	1.979	5.912	1.756
Social isolation	2.636	1.148	2.038	1.007
Defectiveness	2.177	0.920	1.593	0.726
Failure	2.239	1.217	1.654	0.925
Dependence	2.245	1.012	1.696	0.850
Vulnerability	2.953	1.269	2.326	1.134
Enmeshment	2.465	1.054	2.186	1.053
Subjugation	2.734	1.200	2.093	0.980
Self-sacrifice	3.883	1.102	3.548	1.371
Recognition seeking	2.596	1.222	2.142	1.105
Emotional inhibition	2.872	1.369	2.344	1.183
Unrelenting standards	3.380	1.247	2.941	1.270
Pessimism	2.949	1.284	2.513	1.251
Punitiveness	3.138	1.139	2.704	1.148
Entitlement	2.874	1.141	2.554	1.143
Insufficient self-control	2.582	1.187	2.227	1.085

The MANOVA analysis was done to investigate the influence of types of paternal parenting on cognitive patterns: a factorial design of 4 types (affectionate constraint, optimal, affectionless control, and neglectful parenting) × 18 (cognitive schemas), but types of paternal parenting do not seem to affect any cognitive schema (Wilks's Lambda = 325.59; *p* < 0.16).

Likewise, the level of paternal care does not seem to affect any cognitive schemas; although the univariate analysis of variance (ANOVA one-way) reveals the influence of the level of the father's overprotection on: the tendency to social isolation [*F*_(1, 129)_ = 4.89; *p* < 0.05] and the sense of defectiveness [*F*_(1, 129)_ = 4.01; *p* < 0.05]. The analysis of the average scores shows that (see Table 8): the higher level of the father's control influences the tendency to social isolation and defectiveness. These data seem to confirm the second research hypothesis.

**Table 8:** **Means and standard deviation of cognitive schemas with respect to the level of father's overprotection in the target group**.

**Measures**	**Low level of overprotection**		**High level of overprotection**	
	***M***	***SD***	***M***	***SD***
Emotional deprivation	2.508	1.155	2.706	1.438
Abandonment	2.922	1.015	3.101	1.095
Mistrust	6.285	1.888	6.624	2.041
Social isolation	2.091	0.987	2.514	1.184
Defectiveness	1.709	0.750	1.999	0.947
Failure	1.852	1.004	1.983	1.192
Dependence	1.822	0.859	2.060	1.044
Vulnerability	2.615	1.319	2.607	1.157
Enmeshment	2.179	1.051	2.438	1.059
Subjugation	2.267	1.126	2.494	1.132
Self-sacrifice	3.719	1.385	3.682	1.146
Recognition seeking	2.201	1.091	2.486	1.245
Emotional inhibition	2.586	1.414	2.582	1.178
Unrelenting standards	3.210	1.349	3.075	1.215
Pessimism	2.707	1.253	3.075	1.215
Punitiveness	2.941	1.114	2.863	1.208
Entitlement	2.726	1.156	2.674	1.151
Insufficient self-control	2.370	1.958	2.41	1.223

In reference to the control group, a MANOVA analysis shows the influence of the level of maternal care (*p* < 0.001) and maternal control (*p* < 0.001) on all cognitive schemas (see Table 9). Likewise, the level of paternal care (*p* < 0.001) and paternal control (*p* < 0.001) seems to affect all cognitive schemas (see Table 10).

**Table 9:** **The breakdown of the univariate effects with respect to the level of maternal parenting in the control group**.

**Measures**	**Maternal Care**		**Maternal protection**	
	***F***	***P-value***	***F***	***P-value***
Emotional deprivation	52.787	<0.001	65.002	<0.001
Abandonment	18.720	<0.001	20.763	<0.001
Mistrust	26.697	<0.001	42.041	<0.001
Social isolation	46.245	<0.001	57.160	<0.001
Defectiveness	17.968	<0.001	43.001	<0.001
Failure	21.726	<0.001	16.131	<0.001
Dependence	24.039	<0.001	24.388	<0.001
Vulnerability	22.387	<0.001	10.201	<0.001
Enmeshment	82.496	<0.001	88.933	<0.001
Subjugation	38.586	<0.001	98.268	<0.001
Self-sacrifice	8.371	<0.001	15.543	<0.001
Recognition seeking	17.277	<0.001	43.932	<0.001
Emotional inhibition	20.578	<0.001	27.713	<0.001
Unrelenting standards	14.571	<0.001	9.083	<0.001
Pessimism	22.734	<0.001	33.442	<0.001
Punitiveness	17.810	<0.001	17.596	<0.001
Entitlement	17.228	<0.001	29.065	<0.001
Insufficient self-control	15.527	<0.001	40.222	<0.001

**Table 10:** **The breakdown of the univariate effects with respect to the level of paternal parenting in the control group**.

**Measures**	**Paternal care**		**Paternal protection**	
	***F***	***P-value***	***F***	***P-value***
Emotional deprivation	57.155	<0.001	39.380	<0.001
Abandonment	12.479	<0.001	10.607	<0.001
Mistrust	15.671	<0.001	23.570	<0.001
Social isolation	14.295	<0.001	19.263	<0.001
Defectiveness	14.821	<0.001	9.444	<0.001
Failure	4.235	<0.001	4.694	<0.001
Dependence	8.803	<0.001	4.780	<0.001
Vulnerability	7.423	<0.001	3.518	<0.001
Enmeshment	123.491	<0.001	105.257	<0.001
Subjugation	49.228	<0.001	34.292	<0.001
Self-sacrifice	28.398	<0.001	11.583	<0.001
Recognition seeking	20.276	<0.001	14.272	<0.001
Emotional inhibition	7.779	<0.001	5.596	<0.001
Unrelenting standards	6.948	<0.001	6.364	<0.001
Pessimism	20.169	<0.001	17.851	<0.001
Punitiveness	7.128	<0.001	6.507	<0.001
Entitlement	27.863	<0.001	25.768	<0.001
Insufficient self-control	27.655	<0.001	11.547	<0.001

As previously specified, an analysis of hierarchical regression for separate blocks was used to detect the predictor variables of cognitive schemas in all participants. The multiple regression analysis shows how being an inmate, having a low level of maternal care and an high level of maternal control are predictive of the tendency to social rejection, and they explain 37.1% of the overall variance (see Table 11).

**Table 11:** **Summary of the linear regression analyses predicting social rejection**.

**Model**	**Variable**	***R*^2^**	**Adjusted *R*^2^**	***SE***	***B***	**T**	***P***
1	Offender/Non-offender	0.28	0.277		−0.529	−0.973	< 0.001
2	Offender/Non-offender	0.297	0.289	0.112	−0.525	−0.973	< 0.001
	Age			0.005	−0.075	−1.379	0.169
	Education			0.101	0.115	2.131	0.034
3	Offender/Non-offender	0.297	0.289	0.109	−0.598	−11.451	< 0.001
	Age			0.005	0.096	−1.891	0.060
	Education			0.095	0.093−	1.835	0.068
	Mother's care			0.008	−0.215	−3.940	< 0.001
	Mother's overprotection			0.008	0.159	2.957	0.003
4	Offender/Non-offender	0.389	0.371	0.110	−0.594	−11.250	< 0.001
	Age			0.005	−0.098	−1.926	0.055
	Education			0.096	−0.100	1.944	0.053
	Mother's care			0.008	0.208	−3.769	< 0.001
	Mother's overprotection			0.008	0.141	2.529	0.012
	Father's care			0.005	−0.005	−0.105	0.916
	Father's overprotection			0.007	0.066	1.221	0.223

*β, beta standardized coefficients*.

Moreover, the being an inmate, with young age, reduced maternal care, and high maternal control are predictive of the impaired autonomy, explaining 65.1% of the overall variance (see Table 12).

**Table 12:** **Summary of the linear regression analyses predicting reduced autonomy**.

**Model**	**Variable**	***R*^2^**	**Adjusted *R*^2^**	***SE***	***B***	***T***	***P***
1	Offender/Non-offender	0.626	0.625		0.791	20.215	< 0.001
2	Offender/Non-offender	0.631	0.626	0.112	0.794	20.306	< 0.001
	Age			0.005	−0.063	−1.615	0.108
	Education			0.101	0.023	0.594	0.553
3	Offender/Non-offender	0.659	0.652	0.109	0.755	19.423	< 0.001
	Age			0.005	−0.077	−2.025	0.044
	Education			0.095	0.014	0.371	0.711
	Mother's care			0.008	−0.084	−2.069	0.040
	Mother's overprotection			0.008	0.129	3.208	0.002
4	Offender/Non-offender	0.661	0.651	0.110	0.762	19.363	< 0.001
	Age			0.005	−0.080	−2.093	0.037
	Education			0.096	0.013	0.349	0.727
	Mother's care			0.008	−0.085	−2.074	0.039
	Mother's overprotection			0.008	0.121	2.907	0.004
	Father's care			0.005	−0.037	0.961	0.337
	Father's overprotection			0.007	0.029	0.728	0.467

*β, beta standardized coefficients*.

An high level of paternal overprotection (β = 0.13, *p* < 0.01) is the only predictor of the other-directedness domain in the group of participants (0.5% of the overall variance explained); only the young age (β = −0.21, *p* < 0.001) is a predictor of the impaired limits domain (0.7% of the overall variance explained).

Finally, only young age (*F* = −0.16; *p* > 0.01), and high level of maternal control (*F* = 0.18; *p* < 0.05) seem to be predictors of the overvigilance domain (25.3% of the overall variance explained).

## Conclusion and discussion

This work partially disconfirmed the literature, according to which adult male offenders had a dysfunctional attachment with both parents; in actual fact, the target group have an affectionate constraint attachment with their mother, whereas, one third of the population has a negligent attachment with their father.

Comparing the target and control groups, it was possible to evaluate whether adult offenders presented different cognitive schemas when compared to non-offenders with similar socio-demographic characteristics. Outcomes confirmed that in the control group all schemes had lower scores than cut off scores; but the target group reported an instability of those available for support and connection (significant others); expectations that others would hurt, abuse, and humiliate; hypercriticalness toward themselves and others and the tendency to sacrifice for others.

The results, according to the literature and to the research hypothesis, show that in the target group: the affectionless control maternal parenting seems to affect the social-relational ability of participants, characterized by dependence on another person and enmeshment, or the fear of the other and subjugation, resulting in vulnerability and the fear of being exploited and abandoned, leading the subject to emotional-affective inhibition and socio-relational isolation.

More in detail, in the offender group, the reduced maternal care appears to negatively influence the perception that the subject has of himself, in terms of emotional deprivation, abandonment, mistrust, vulnerability, emotional inhibition, and punitiveness, all of which reach dysfunctional scores. Moreover, the perception of excessive paternal control influences the tendency to social isolation and the presence of a sense of defectiveness. In reference to the paternal parenting, the data show that the higher level of paternal control influences the tendency to social isolation and defectiveness.

Our study underlines that being an inmate with maternal parenting characterized by affectionless control is predictive of the tendency to expect that own needs for stability, nurturance, security, and empathy in family relationships will not be met in a consistent or predictable way, and consequently it is predictive of the difficulty in separating and functioning independently.

Furthermore, the condition of prisoner together with the high paternal over-control are predictive of the tendency to other-directedness, pattern widely present among the prisoners involved in the research.

These results confirm the Young's model that (2003) emphasizes the role of parents in the development of the Early Maladaptive schemas. Young, also, hypothesizes that the loss of autonomy and the high level of control lead to the development of severe schemas, emphasizing the role of the family in the development of such schemas.

So, the loss of autonomy in the subjects analyzed in this study comes from the family, and also from factors outside the family such as the condition of being an inmate. This kind of loss of autonomy as well as the general prevailing insecurity could very well lead to additional Early Maladaptive Schemas.

Therefore, with the use of control group it was possible to test whether the observed relationship between the psychological constructs can be specific for offenders, or it can be found in general population. In fact, although in both groups (offenders and non-offenders) parenting influenced the cognitive schemas, in the control group all schemes had lower scores than cut off scores, and in the target group emerged high scores in the disconnection and rejection domain, in the over-vigilance and the other-directedness domains. These results suggest that a high level of parental control may be related to the offending behavior generally, and that certain combination of over-protection, and severe cognitive schemes may relate more specially to offending behavior.

Although, both dimensions of parenting, care, and control, seem to be important, few studies have focused on these styles of parenting and only a small part of the literature has investigated the role of the father and the effect that the lack of paternal support has on cognitive patterns of the children when they become adults.

The present study had a number of limitations that need to be considered. First of all, the study measures the relationship between parenting and cognitive schemas in a group of male adult offenders, so, as is indubitably the case in all correlation studies, the results in no way allow for casual conjectures.

Also, the use of only an explicit measure of cognitive schemas; infact, although, the Italian version of the YSQ is validated, future studies should add alternatives to self-report. Also, we did not use a measure to account for socially desirable responding and/or the tendency to minimize pathology.

Another limitation was the small sample size in this study which may have limited the ability to detect further differences in Maladaptive Schemas; thus, the generalizability of our findings remains unclear.

It might have been useful to take some other covariates into account, for example social integration before the crime and personality structure level.

So, an appropriate research design would ideally include a prospective cohort study of risk population because it would be less subject to bias and it would allow for an estimation of the incidences, although it would also be time-consuming.

## Author contributions

The authors MP and GC have made substantial, direct and intellectual contribution to the work; the author YT has only taken care of the administration of the questionnaires. All authors approved it for publication.

### Conflict of interest statement

The authors declare that the research was conducted in the absence of any commercial or financial relationships that could be construed as a potential conflict of interest.
